# Reduced global longitudinal and radial strain with normal left ventricular ejection fraction late after effective repair of aortic coarctation - a CMR feature tracking study

**DOI:** 10.1186/1532-429X-14-S1-O59

**Published:** 2012-02-01

**Authors:** Shelby Kutty, Sheela Rangamani, Jeeva Venkataraman, Ling Li, Andreas Schuster, Scott E Fletcher, David A Danford, Philipp B Beerbaum

**Affiliations:** 1 Joint Division of Pediatric Cardiology, University of Nebraska College of Medicine/ Creighton University School of Medicine, Children’s Hospital and Medical Center, Omaha, NE, USA; 2 King's College London British Heart Foundation Center of Excellence, National Institute of Health Research Biomedical Research Center at Guy's and St. Thomas' NHS Foundation Trust, Division of Imaging, London, UK; 3 Depts for Radiology and Paediatric Cardiology, St Radboud Medisch Universiteit, Nijmegen, Netherlands

## Background

Little is known about the state of left ventricular (LV) mechanics long-term after repair of coarctation of the aorta (COA). We sought to determine whether LV myocardial deformation indices measured by cardiac magnetic resonance-based feature tracking (CMR-FT) were abnormal in repaired COA with normal LV ejection fraction (EF), relative to healthy adult controls, and whether such alteration was related to LV hypertrophy.

## Methods

We retrospectively identified 81 patients after COA repair (31 female male; age 25±8.5 years) with inclusion criteria at follow-up CMR of: age ≥13years, time post-repair ≥10 years, no aortic valve disease, LV-EF>50%). CMR-FT was performed in four-chamber and short-axis steady-state free-precession cine sequences to derive global and segmental LV deformation indices and indexed (body surface area, BSA) end-diastolic/end-systolic volumes, mass, and LV-EF. LV deformation and volumetric indices were compared firstly between COA patients and normal controls (n=20, 10 female, age 37±7 years), and secondly between COA patients with (mass >97th percentile for gender and BSA) versus without LV hypertrophy.

## Results

In COA patients versus healthy controls, LV-EF was 62 ±7.2 % (mean±SD) versus 58±3.0% (p=0.01), and LV mass 66±16.8 g/m2 versus 57.7±6.0 g/m2 (p=0.0001). The LV global longitudinal strain (GLS) was decreased to -17.0±4.7% in COA versus -20±5% in controls (p=0.02) and the global radial strain (GRS) was reduced to 40±15% versus 50±12.4% in controls (p=0.003). In contrast, the global circumferential strain (GCS) was preserved in COA patients at -23±4.7% versus -24.6±2.4% in controls (p=0.14). Regionally, decrease in LS was particularly marked in the basal segments (septal, p=0.005 and lateral, p=0.013). In COA with LV hypertrophy (n=45, LV mass 76.3±12.8) versus COA without LV hypertrophy (n=36, LV mass 52.2±10), GLS was more markedly decreased to -15.7±4.8% versus -18.5±4.2%, p= 0.016). In contrast, both GRS and GCS were similar between both COA subgroups(p=0.49 and 0.27).

## Conclusions

In patients late after COA repair with normal LV-EF, GLS and GRS are reduced whilst GCS is preserved, compared with controls. GLS is even more reduced in the presence of LV hypertrophy. Longitudinal strain is particularly reduced in basal LV segments. GLS may qualify as indicator of early LV dysfunction if confirmed in prospective outcome studies.

## Funding

None.

